# The development and excretion of *Toxoplasma gondii* oocyst manipulate the gut microbiota in its definitive host

**DOI:** 10.1186/s13071-025-06925-6

**Published:** 2025-07-09

**Authors:** Gui-Hua Zhao, Bei-Bei Zhou, Zhi-Heng Cao, Ting Xiao, Ya-Nan Li, Wen-Ju Zhu, Hang Sun, Huan-Huan Xie, Xiao-Man Xie, Jun-Mei Zhang, Qi Wang, Xin Zhang, Jin-Jing Xie, Hong-Jie Dong, Chao Xu, Kun Yin

**Affiliations:** 1 Shandong Institute of Parasitic Diseases, Shandong First Medical University & Shandong Academy of Medical Sciences, Jining, 272033 China; 2 School of Public Health, Shandong First Medical University & Shandong Academy of Medical Sciences, Jinan, China; 3 Jining Rencheng District Center for Disease Control and Prevention, Jining, China

**Keywords:** *Toxoplasma gondii*, Oocyst excretion, Cat, Gut microbiota, Function and composition

## Abstract

**Background:**

Oocysts serve as the primary source of *Toxoplasma* infection. Therefore, understanding oocyst development and exploring effective strategies to prevent oocyst excretion are crucial for controlling toxoplasmosis.

**Methods:**

In this study, shotgun metagenomics was employed to characterize the functional and compositional changes in the gut microbiota of cats during oocyst development. The Spearman correlation test was utilized to analyze the correlation between differential Kyoto Encyclopedia of Genes and Genomes (KEGG) pathways and carbohydrate-active enzymes (CAZymes) in key bacteria regulating oocyst excretion.

**Results:**

The results revealed that group A (sexual initiation stage) displayed a lower number of functional genes, which were restored to normal levels in group B (oocyst excretion stage), compared with group C (*Toxoplasma*-uninfected samples). The abundance of 39 KEGG pathways, 106 CAZymes, and 98 virulence factors (VFs) varied significantly among the three groups. The atrazine degradation pathway, associated with sexual development, was upregulated in group B. CAZymes involved in restoring the intestinal mucosal barrier and VFs related to iron metabolism, antibiotic resistance, and suppression of host immunity were enriched in group B. Sexual initiation and oocyst excretion resulted in reduced gut bacterial diversity and microbiota dysbiosis. Probiotics and bacteria related to linoleic acid (LA) uptake were dominant in both group A and group B. *Bacteroides stercoris* was the most significantly upregulated bacterium and could influence the expression of carbohydrate-binding modules (CBMs) and glycoside hydrolases (GHs) in group B.

**Conclusions:**

During the oocyst development/excretion stage, the function and composition of the cat gut microbiota changed significantly. In addition, *Bacteroides stercoris* may play a crucial role in oocyst excretion by regulating key candidates of CBMs and GHs. Our findings lay the foundation for investigating the regulatory mechanisms of oocyst excretion.

**Graphical Abstract:**

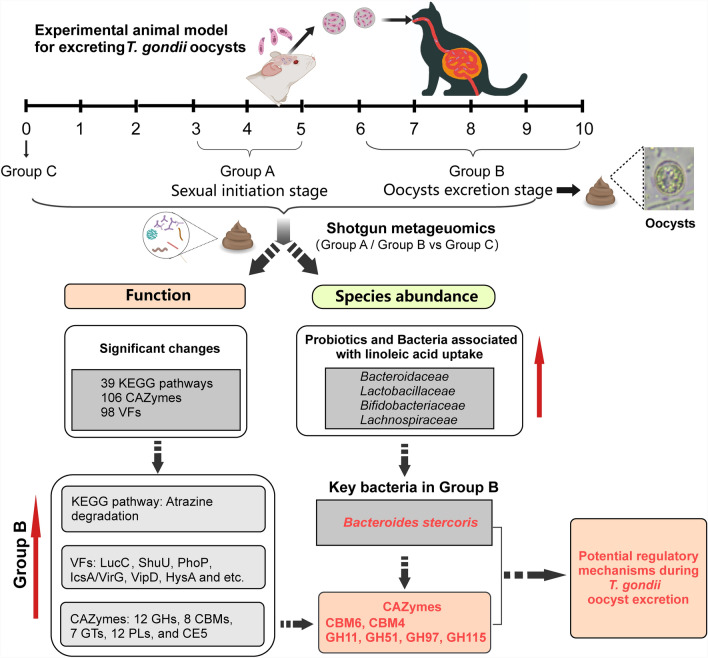

## Background

*Toxoplasma gondii* (*T. gondii*), regarded as one of the most successful parasites, is capable of infecting any warm-blooded animal. It is estimated that approximately 30% of the global population is infected with *T. gondii* [1]. The seroprevalence of *T. gondii* can exceed 80% in both humans and animals in some regions, such as Egypt, Brazil, and France [2, 3]. In China, there has been an increasing trend in seroprevalence rates, with a prevalence ranging from 2.3% to 35.6% in humans and 1.3% to 82.7% in animals [4]. *T. gondii* infection brings serious threats to both human health and livestock production. In Brazil, the prevalence of congenital toxoplasmosis ranges from 0.4 to 2 cases per 1000 newborns [5], and approximately one fifth of infected individuals experience persistent visual impairment [6, 7]. *Toxoplasma* infection in Egypt has been linked to increased mortality rates among pediatric patients diagnosed with brain tumors [8]. Recent studies have also revealed a correlation between chronic *Toxoplasma* infection and mental disorders in humans, including schizophrenia, depression, suicidal behavior, and obsessive–compulsive disorder [9–12]. Consequently, toxoplasmosis has become an important global public health concern.

Oocysts, excreted into the environment, play an important role in the transmission of toxoplasmosis. They serve as the main source for *T. gondii* infection among aquatic and semi-aquatic animals [13]. All outbreaks of toxoplasmosis in Brazil have been attributed to oocysts since 2000 [14]. Although most adult infections occur via ingestion of tissue cysts from infected meat, children in Europe also become infected through oocysts. In addition, oocysts contribute to outbreaks of toxoplasmosis in pigs and an increase in the mortality rate of immunocompromised individuals in China [15].

Cats, one of the closest companions to humans, are also the most important definitive hosts of *T. gondii*, with a wide range of seropositivity varying from 2.2% to 100% [16]. Within 1–3 weeks of primary *T. gondii* infection, they can shed up to 20 million oocysts per day for 10–20 days in their feces. It is noteworthy that infected feral cats may intermittently shed oocysts throughout their lifespan [17]. Moreover, oocysts are stable for up to 18 months under unfavorable environmental conditions and resistant to many chemical disinfectants [18]. Infectious oocysts can contaminate water sources, food, and soil. Surprisingly, even a single oocyst has the potential to induce infection in animals [19].

Therefore, exploring effective strategies to prevent oocyst excretion is crucial for controlling toxoplasmosis. It is well known that *T. gondii* can only initiate sexual reproduction in feline intestines; thus, the feline gut microbiota may have key correlations with a successful initiation of sexual reproduction, as well as the oocyst excretion process. There are also previous studies showing that other intestinal protozoan parasites, such as *Cryptosporidium* and *Giardia*, could cause significant alterations in the host gut microbiota function and composition, thereby modulating the progression of protozoan infection and the outcome of parasitic disease [20–24]. However, up to now, few studies on the alterations of the gut microbiota of *T. gondii* definitive hosts have been reported.

Thus, in the present study, we hypothesized that the gut microbiota composition and functions of *T. gondii’s* definitive host may be significantly altered to facilitate parasite excretion during the sexual reproduction process. Therefore, we have selected the highly prevalent *Chinese I TgCtwh6* strain to infect Chinese fox tabby cats, establishing an animal model for *Toxoplasma* oocyst excretion. Shotgun metagenomics was used to investigate variations in the gut microbial function and composition during the sexual initiation phase and the oocyst excretion phase, to explore potential key bacteria affecting oocyst development. This study has revealed the impact of *Toxoplasma* oocyst development on cat gut microbiota and laid a fundamental basis for investigating the regulatory mechanism of oocyst excretion.

## Methods

### Animals and parasite strains

Cats: Female fox tabby cats from southwest China, known for their relatively docile nature, were chosen as the experimental animals in this study. The experimental cats had been housed in our laboratory under identical conditions, provided with commercial feed and clean water, for an extended period. Second-generation cats, aged 3 months, were confirmed to be *T. gondii*-negative, free of feline immunodeficiency virus and other gastrointestinal pathogens. These cats were subsequently selected to serve as the infection models that could excrete *Toxoplasma* oocysts and were maintained on qualified commercial feed and sterile water in a specific pathogen-free (SPF) environment.

Mice: Specific pathogen-free (SPF) Kunming (KM) mice, aged 6–8 weeks, were obtained from Jinan Pengyue Laboratory Animal Breeding Co., Ltd. and were used to continuously culture and passage *T. gondii* cysts, providing parasites to facilitate *Toxoplasma* sexual reproduction in cats.

Parasite strain: *TgCtwh6* strain (*Chinese I*) was donated by the Anhui Provincial Key Laboratory of Pathogen Biology, Anhui Medical University, and preserved at the Control Central Laboratory of Shandong Institute of Parasitic Disease.

### Establishment of *T. gondii* sexual reproductive animal model

Establishment and identification methods of sexual/oocyst development in a cat model infected with *TgCtwh6* strain have been previously described in our published literature [25]. Specifically, KM mice were orally infected with the *TgCtwh6* strain (30 cysts each), and brain tissue cysts were collected 42 days postinfection. After counting, a total of 600 brain tissue cysts were administered orally to each cat. The infected cats were housed separately and fed the same diet of commercial feed and clean water to ensure they were not exposed to other pathogens during the oocyst development period. Their fecal samples were collected daily, dissolved in a 2% H_2_SO_4_ solution, and filtered through a 30-mesh filter to remove large debris. Then oocysts were examined under a microscope. Meanwhile, DNA of oocysts in fecal samples was extracted using the QIAamp Fast DNA Stool Mini Kit (Qiagen) and identified through polymerase chain reaction (PCR) targeting *T. gondii*-specific genes (529 base pairs (bp) repetitive sequence). The primers of PCR were as follows: forward 5-CGCTGCAGGGAGGAAGACGAAAGTTG-3 and reverse 5-CGCTGCAGACACAGTGCATCTGGATT-3.

### Design of experimental groups

**Fig. 1:**
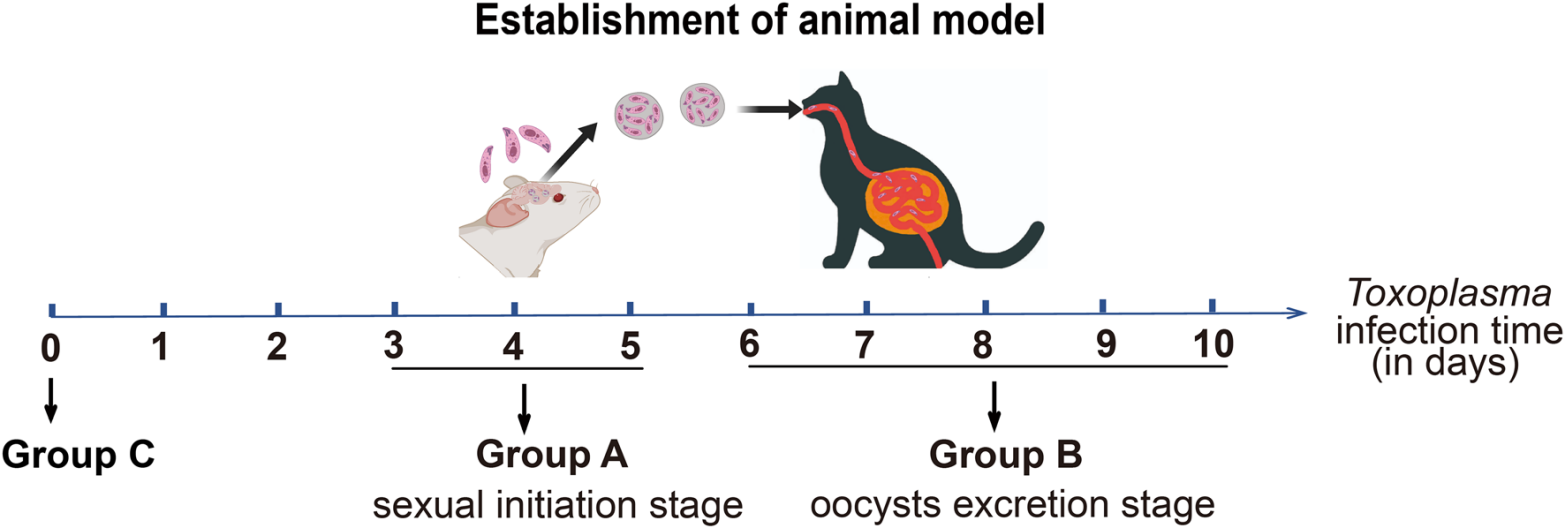
The design of the experimental groups

The sexual cycle of *T. gondii* was divided into two distinct stages: the sexual initiation stage (pre-sexual stage) and the oocyst excretion stage (sexual stage). These stages were treated as independent experimental groups to investigate the impact of oocyst development on the cat gut microbiota. Results of our previous study indicated that oocysts were absent in cats feces, while schizonts were observed within the cat intestinal epithelial cells (IECs), during the sexual initiation stage (3–5 days postinfection) [25]. Oocysts were subsequently observed in cats’ feces during the oocyst excretion stage (5 and 10 days postinfection) [25]. Therefore, three 3-month-old healthy cats were orally inoculated with *TgCtwh6* cysts in this study. Fecal samples were collected from the three cats at three different developmental time points: 0 days postinfection (uninfected stage, designated as the control group C), 3–5 days postinfection (sexual initiation stage, designated as group A), and 5–10 days postinfection (oocyst excretion stage, designated as group B). The design of experimental groups is illustrated in Fig. 1. The collected fecal samples were used to characterize the functional genes and the species composition of the gut microbiota using shotgun metagenomics.

### Fecal sample collection

The middle portion of fresh cat feces was aseptically collected using sterile samplers to ensure uniformity in sampling conditions between the experimental and control groups. Subsequently, the collected samples were divided into 0.5–2-g aliquots before being transferred into frozen tubes. These tubes were rapidly transferred to liquid nitrogen for 3 h and then stored at −80 ℃ in a refrigerator until use.

### Illumina library preparation and sequencing

The total genomic DNA was extracted from the fecal sample (500 mg) using the PowerSoil DNA Isolation Kit (MO BIO) according to the manufacturer’s instructions. Subsequently, the concentration and purity of extracted DNA were analyzed using the Qubit dsDNA HS Assay Kit on a Qubit 3.0 fluorometer (Invitrogen), and its integrity and fragment size distribution were assessed on a 1% agarose gel electrophoresis. The AHTS Universal Plus DNA Library Prep Kit was used to construct the library, following the manufacturer’s recommendations, with index codes added for sequence annotation in each sample. Firstly, the DNA was fragmented through enzyme digestion, followed by end repair, and then sequencing adapters were ligated to the 3′ ends. The resulting ligation products were purified using Vazyme DNA Clean Magnetic Beads and sieves obtained from film screening. Amplification of purified product was carried out using PCR Primer Mix 3 and VAHTS HiFi Amplification Mix, followed by secondary purification with Vazyme DNA Clean Magnetic Beads. The library insert size was evaluated on the Qsep-400, while its concentration was determined using Qubit 3.0. Finally, sequencing of libraries took place on the Illumina NovaSeq6000 platform (San Diego, CA, USA) following a PE150 strategy along with reagents from the NovaSeq 6000 Reagent Kit.

### Construction of non-redundant gene sets

The Trimmomatic (version 0.33) was used to filter raw reads to obtain high-quality sequencing data (clean reads). Subsequently, Bowtie2 software (version 2.2.4) was utilized to align these clean tags with the host genome sequence and eliminate any host contamination. The genome was subsequently assembled using the MEGAHIT software (version 1.1.2), excluding contig sequences shorter than 300 bp in length. Finally, the QUAST software (version 2.3) was used for comprehensive evaluation of the assembly results obtained. Gene-coding regions were identified using Meta GeneMark software (http://exon.gatech.edu/meta_gmhmmp.cgi, version 3.26). To construct a non-redundant gene set, MMseqs2 software (https://github.com/soedinglab/mmseqs2, version 11-e1a1) was applied with a similarity threshold of 95% and a coverage threshold of 90%.

### Function annotation

The amino acid sequences of non-redundant gene sets were aligned with the KEGG database using Diamond (version 0.9.24) and annotated with gene ontology (GO) terms using Blast2GO (version 2.5). Subsequently, the non-redundant gene sets were queried against the Carbohydrate-Active Enzymes (CAZy) database using HMMER (version 3.0), the Comprehensive Antibiotic Database (CARD) using RGI (version 4.2.2), the Virulence Factors of Pathogenic Bacteria database (VFDB) setA and setB using BLASTP (version 2.2.31+) with an *e*-value threshold of < 1e−05.

### Gut microbiota diversity analysis

#### Function diversity analysis

The composition and relative abundance of KEGG pathways, CAZymes, antibiotic resistance ontologies (AROs), and virulence factors (VFs) were statistically analyzed for each sample on the basis of gene function annotation results. Principal component analysis (PCA) and analysis of similarities (ANOSIM) were employed to compare the beta diversity and functional differences of the intestinal microbiota among different groups on the basis of KEGG, CAZy, CARD, and VFDB databases.

#### Species diversity analysis

The taxonomic composition and relative abundance of species in the samples were determined by aligning non-redundant gene sequences with those from the non-redundant protein database (Nr). Alpha diversity of the intestinal microbiota was assessed using Chao and Shannon indices. Beta diversity was analyzed by PCA and ANOSIM. Feature bacteria serving as biomarkers were identified through Linear discriminant analysis Effect Size (LEfSe).

### Statistical analysis

Metagenomic sequencing data were acquired using the Illumina HiSeq PE250 system. Wilcoxon tests were utilized to evaluate variations in multiple variables among different groups. Principal coordinates analysis (using Bray–Curtis distance) was conducted using the vegan package. ANOSIM was performed utilizing the vegan R package. The correlation between KEGG pathways and CAZymes of key bacteria was examined through the Spearman correlation test. The statistical significance level was set at *P* < 0.05 for all analyses.

## Results

### Functional gene changes of the cat gut microbiota at the sexual initiation stage

**Fig. 2:**
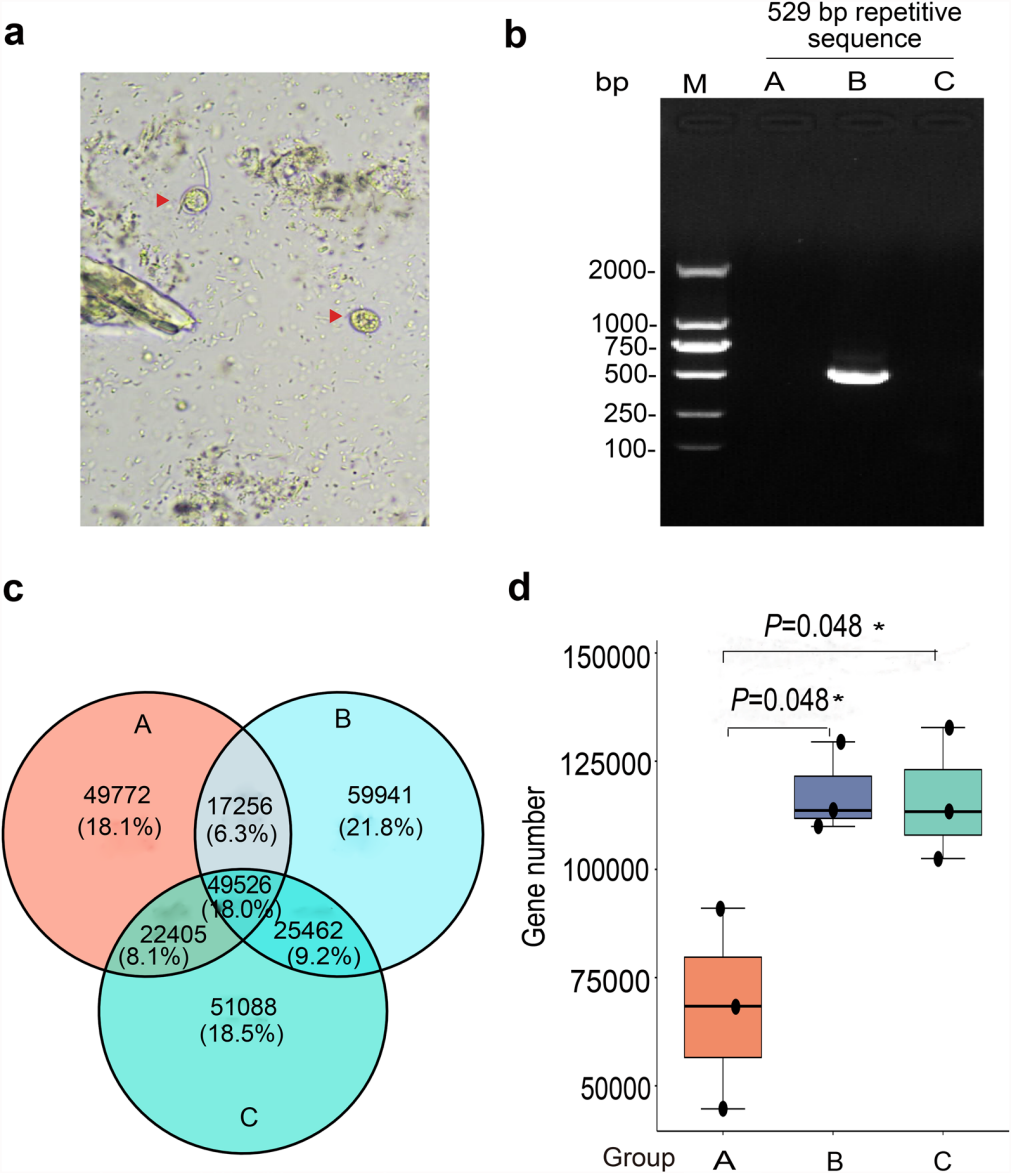
Identification of oocysts and comparative analysis of microbial gene counts in the three groups. **a** Microscopic examination of oocysts in fecal samples, with representative oocysts indicated by red arrows (400×). **b** PCR-based detection of oocysts in fecal samples. M: DNA marker DL2000. A, B, and C represent group A, group B, and group C, respectively. **c** The Venn diagram of non-redundant genes in the three groups. The differently sized, color-coded ovals represent individual groups; overlapping regions indicate the number of shared non-redundant genes, whereas non-overlapping regions represent the number of group-specific non-redundant genes. **d** Boxplot depicting the distribution of non-redundant gene counts across the three groups. The *P*-values derived from *t*-tests are labeled on the connecting lines (*P*-values are not displayed when *P* > 0.05)

Oocysts were observed in the fecal samples of group B through microscope examination, while they were absent in the other two groups (group A and group C) (Fig. 2a). Simultaneously, PCR result of *Toxoplasma*-specific 529 bp repeat sequence was detected in the fecal samples of group B, while it was absent in groups A and C (Fig. 2b). The gene sequence data of gut microbiota was successfully obtained using metagenomic sequencing technology from the three groups. After removing redundant sequences, a total of 319,195 non-redundant genes were acquired, with an average of 35,466 genes per group and an average gene length of 721 bp. The non-redundant gene counts for each group were depicted in Fig. 2c. Compared with group C, group A exhibited a substantial decrease in the function genes, whereas group B was restored to a level comparable to group C (Fig. 2d).

### KEGG pathway analysis of the cat gut microbiota during the oocyst development process

**Fig. 3:**
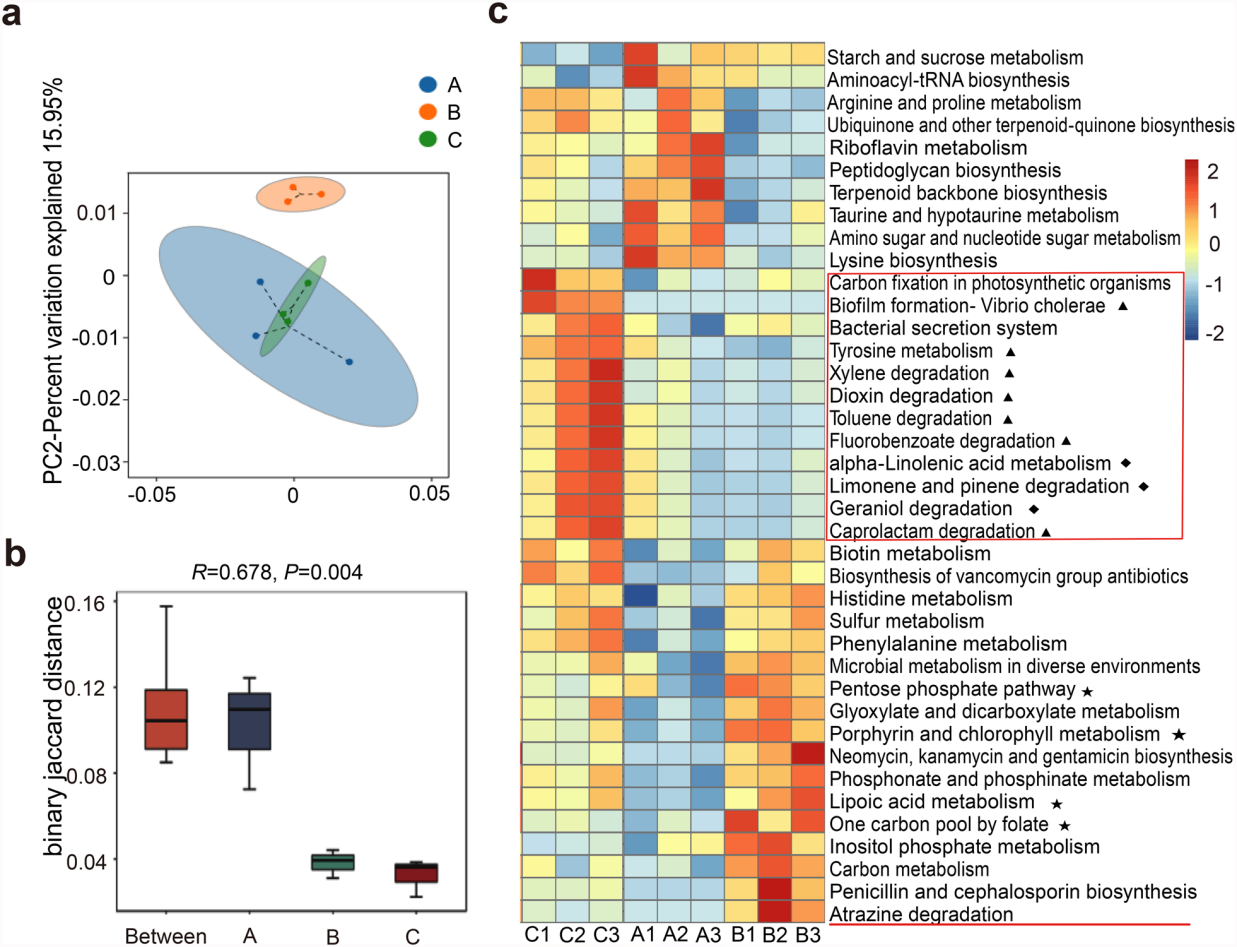
Functional diversity of KEGG pathways in the gut microbiota. **a** PCA of KEGG pathways profiles. Each dot represents an individual sample, with color-coded dots indicating different experimental groups. The *x*-axis displays the first principal component, and the percentage indicates its contribution to sample differentiation. The *y*-axis shows the second principal component, and the percentage represents its contribution to sample differentiation. **b** ANOSIM of KEGG pathways. “Between” refers to the beta distance of samples between all groups, and the following sets represent beta distance within each group. An *R* value close to 1 indicates that inter-group differences are greater than intra-group variations. A *P* < 0.05 indicates statistically significant separation among groups. **c** Heatmap depicting the relative abundance of differentially enriched KEGG pathways. Only pathways with *P* < 0.05 based on parametric differential abundance testing are shown; red indicates upregulation, and blue indicates downregulation of pathways

The beta diversity analysis result indicated significant differences in KEGG pathways of gut microbiota among three groups (Fig. 3a and b). A total of 39 KEGG pathways were identified as significantly distinct (Fig. 3c). Compared with group C, KEGG pathways related to starch and sucrose metabolism as well as aminoacyl-tRNA biosynthesis were upregulated in both group A and group B. Conversely, KEGG pathways associated with neural transmission inhibition (indicated by ▲ in Fig. 3c) and the degradation of antibacterial, anti-inflammatory, and antioxidant substances (indicated by ◆ in Fig. 3c) were downregulated. Moreover, group A exhibited upregulation in KEGG pathways linked to antibiotic biosynthesis, cell growth, tissue repair, and mucosal integrity. In contrast, group B showed upregulation in pathways involved in mitigating oxidative damage (indicated by ★ in Fig. 3c) and producing antibiotics targeting gram-negative bacteria. Interestingly, the atrazine degradation pathway, which has been shown to play a crucial role in regulating the development of male eukaryotic animals [26, 27], was also upregulated in group B.

### Functional factor changes of the cat gut microbiota during the oocyst excretion

**Fig. 4:**
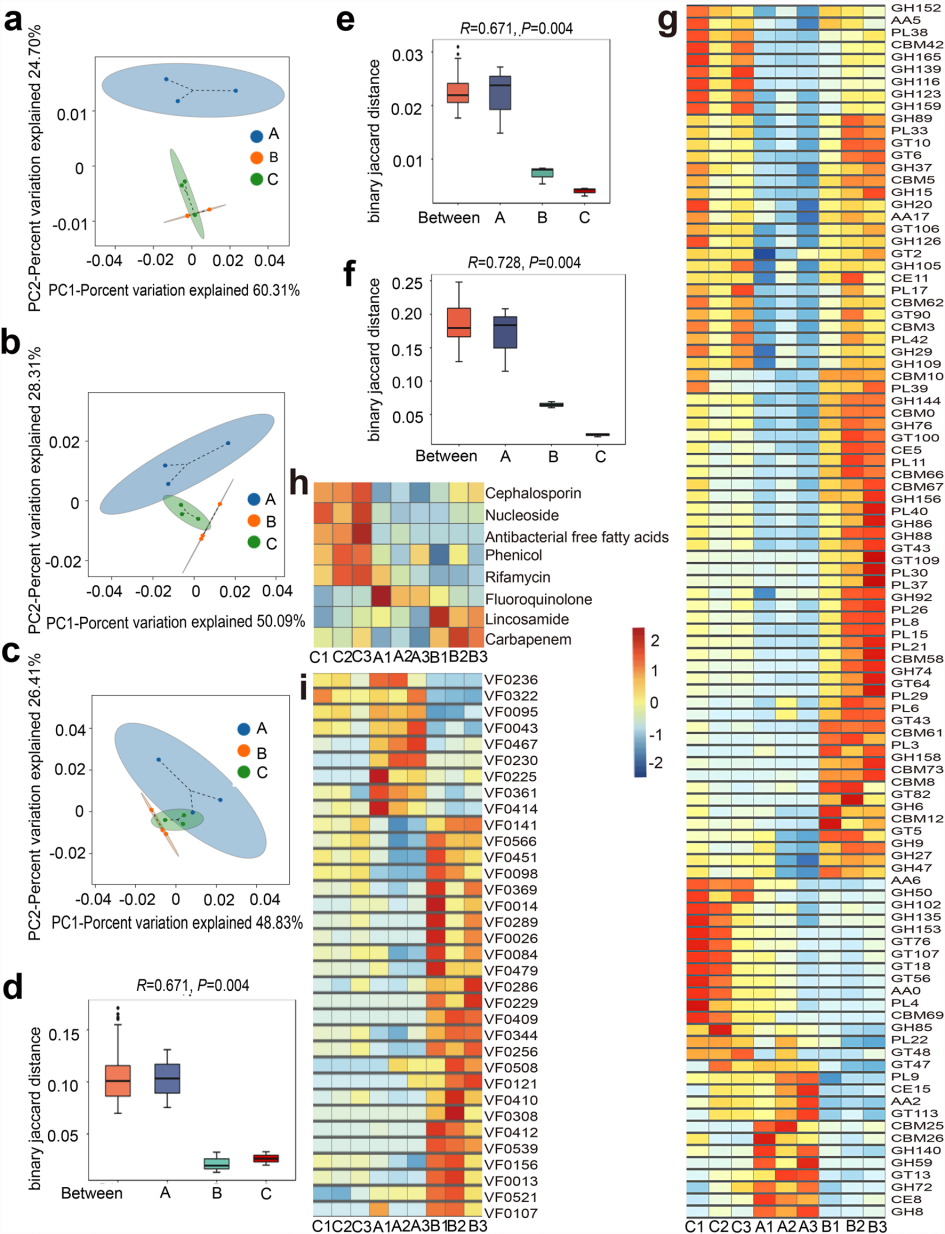
Function diversity of the gut microbiota. **a–c** PCA results based on CAZyme families, AROs, and VFs. Each dot represents an individual sample, with color-coded dots indicating different experimental groups. The *x*-axis displays the first principal component, and the percentage represents its contribution to sample differentiation. The *y*-axis shows the second principal component, and the percentage represents its contribution to sample differentiation. **d–f** ANOSIM results based on CAZyme families, AROs, and VFs. “Between” refers to the beta distance of samples between all groups, and the following sets represent beta distance within each group. An *R* value close to 1 indicates that inter-group differences are greater than intra-group variations. A *P* < 0.05 indicates a statistically significant result. **g** Heatmap showing the relative abundance of 40 CAZyme families significantly enriched in group B. **h** Heatmap depicting the relative abundance of differentially abundant CARDs. **i** Heatmap illustrating the relative abundance of 34 VFs significantly enriched in group A and group B. In the heatmap, red denotes upregulation, and blue indicates downregulation

The beta diversity analysis revealed that the *T. gondii* oocyst development in group A and group B induced substantial alterations in the functional diversity of gut microbiota compared with the control group (group C) (Fig. 4a–c). Specifically, the most significant variation was observed in CAZymes, followed by AROs and VFs (Fig. 4a–c). ANOSIM results indicated that *T. gondii* sexual initiation (group A) significantly affected the function of gut microbiota (Fig. 4d–f).

#### CAZymes

Differential analysis of function genes revealed that the sexual initiation and oocyst excretion significantly affected 106 members of CAZymes within the gut microbiota. Compared with group C, group A exhibited a reduction in several CAZyme families, including GHs, CBMs, and polysaccharide lyases (PLs). However, certain CAZyme members were notably enriched in group A, such as carbohydrate esterases 15 (CE15), AA2, GT113, CBM25, CBM26, GH140, GH59, GT13, GH72, CE8, and GH8 (Fig. 4g). Group B showed a significant enrichment of 40 CAZymes, including 12 GHs, 8 CBMs, 7 glycosyltransferases (GTs), 12 PLs, and CE5 (Fig. 4g). Previous studies demonstrated that these enzymes not only participated in remodeling the intestinal mucosal barrier but also played crucial roles in defending against pathogen invasion [28, 29].

#### AROs

AROs analysis revealed a decrease in the relative abundances of cephalosporins, nucleosides, anti-fatty acids, phenylpropanol, and rifamycin antibiotics, while fluoroquinolone exhibited an increase in both group A and group B. In addition, group B had a higher relative abundance of lincomycin and carbapenem (Fig. 4h).

#### VFs

In the meantime, a total of 98 VFs from groups A and B exhibited significant differences compared with those in group C. Specifically, group A showed a notable enrichment of 9 VFs localized on the bacterial cell membrane, functioning as transporters and receptors (nos. 1–9 in Table 1). Group B showed a significant enrichment of 25 VFs (Fig. 4i, nos. 10–34 in Table 1), which were associated with iron ion metabolism (*iucC*, *shuU*); antibiotic resistance (*ptlG*, *exoU*, *mtrD*); pathogen invasion and intracellular parasitism (*phoP*, *icsA/virG*, *vipD*, *hysA*); immunosuppression (*exsA*, *btpA*, *spvB*, among others); as well as signal transduction (*bepA*).

**Table 1:** Functional analysis of 34 important differential VFs

No.	Gene ID	VFs ID	VFs gene name	VFs name	Function
1	000036647	VF0236	*ompA*	OmpA	Outer membrane protein A
2	000150693	VF0322	*cadF*	CadF	Outer membrane fibronectin-binding protein
3	000119592	VF0095	*pchH*	Pyochelin	ABC transporter ATP-binding protein
4	000155577	VF0043	*bsc3*	Capsule	Polysaccharide biosynthesis protein Bsc3
5	000156563	VF0467	*bauE*	Acinetobactin	Ferric siderophore ABC transporter, ATP-binding protein BauE
6	000161245	VF0230	*iroN*	IroN	Salmochelin receptor IroN
7	000162914	VF0225	*hlyB*	Hemolysin	Hemolysin B
8	000164438	VF0361	*cpsG*	Capsule	MurB family protein
9	000175843	VF0414	*ricA*	RicA	Rab2 interacting conserved protein A
10	000185057	VF0141	*capA*	Capsule	CapA, required for poly-gamma-glutamate transport
11	000191443	VF0566	*fimF*	Type I fimbriae	Type 1 fimbrial minor component
12	000192293	VF0451	*mtrD*	MtrCDE	Multiple transferable resistance system protein
13	000219954	VF0098	*exoU*	ExoU	Type III secretion system effector ExoU, phospholipase A2 activity
14	000258325	VF0369	*bepA*	VirB/VirD4 type IV secretion system	Bartonella effector protein BepA
15	000262505	VF0014	*N*-deacetylase	Intercellular adhesion proteins	Synthesis
16	000314238	VF0289	*mgtC*	MgtC	Possible Mg2 + transport P-type ATPase C MgtC
17	000011155	VF0026	*ptlG*	Ptx	Ptl type IV secretion system VirB10 homolog protein PtlA
18	000043023	VF0084	*xcpR*	XCP	General secretion pathway protein E
19	000059275	VF0479	*exsA*	T3SS	Type III secretion system transcriptional regulator
20	000059946	VF0286	*phoP*	PhoP	Possible two-component system response transcriptional positive regulator PhoP
21	000078899	VF0229	*iucC*	Aerobactin	Aerobactin siderophore biosynthesis protein IucC
22	000242704	VF0409	*vtrA*	T3SS2	Type III secretion system transcriptional regulator
23	000247743	VF0344	*cdsN*	TTSS	Type III secretion system ATPase
24	000252296	VF0256	*shuU*	Shu	Permease of iron compound ABC transport system
25	000112991	VF0508	*yapK*	YapK	Autotransporter protein YapK
26	000122614	VF0121	*icsA/virG*	IcsA (VirG)	Autotransporter, actin tail assembly protein IcsA/VirG
27	000123254	VF0410	*llsG*	LLS	ABC transporter ATP-binding protein LlsG
28	000093371	VF0308	*mbtM*	FadD33	Probable fatty acyl-AMP ligase MbtM
29	000116805	VF0412	*btpA*	BtpA/Btp1/TcpB	Tir domain containing protein BtpA
30	000305479	VF0539	*ecbA*	EcbA	Collagen binding MSCRAMM, EcbA
31	000308785	VF0156	*vipD*	Dot/Icm	Dot/Icm type IV secretion system effector VipD, Phospholipase A1
32	000151104	VF0013	*hysA*	Hyaluronate lyase	Hyaluronate lyase precursor
33	000182267	VF0521	*eccA3*	ESX-3	Type VII secretion system protein EccA3
34	000077725	VF0107	*spvB*	Spv	Type III secretion system effector SpvB, ADP-ribosylation activity

### Key bacterial taxa analysis of the cat gut microbiota during the sexual reproduction stage

#### The diversity of bacterial composition analysis

**Fig. 5:**
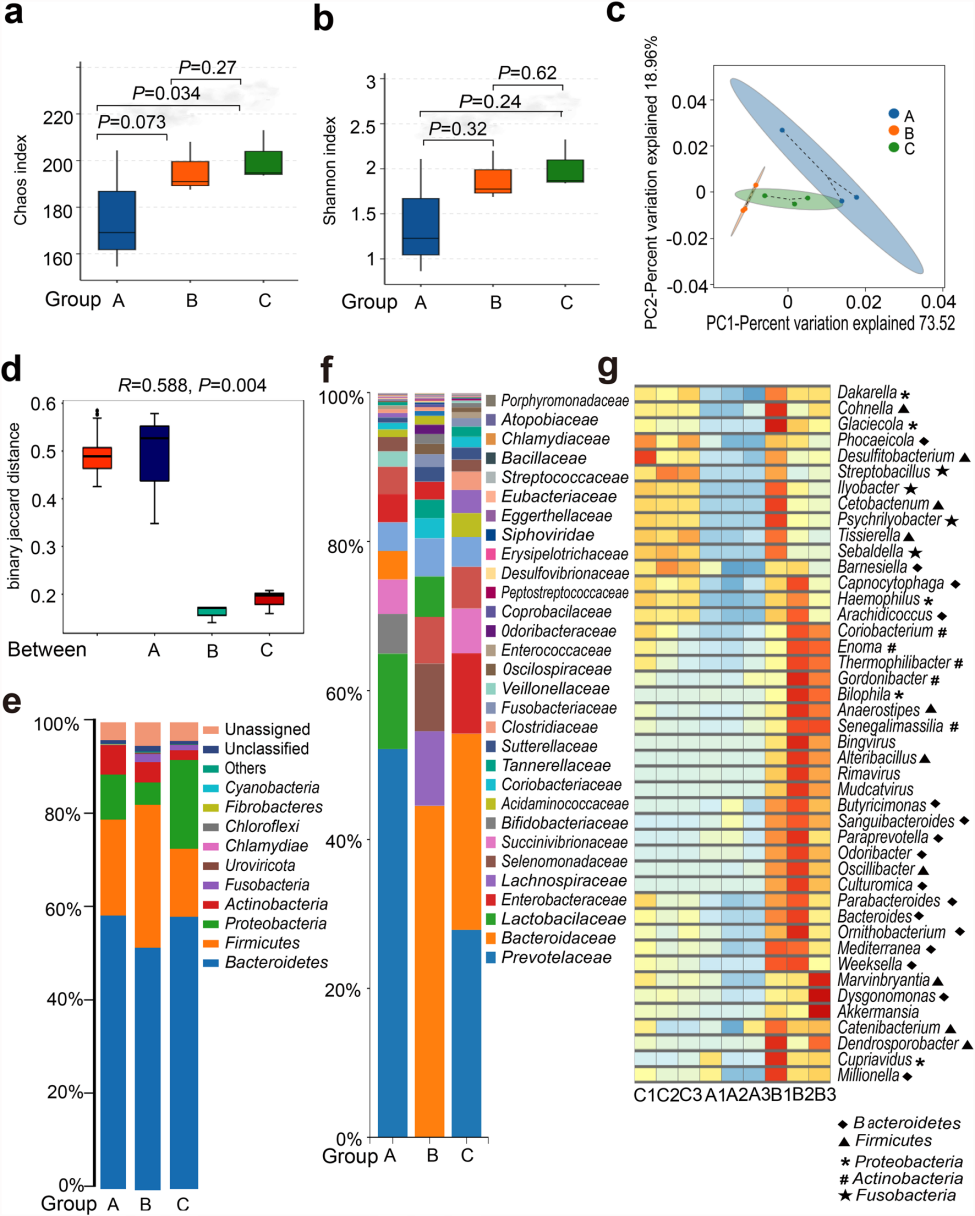
The diversity and composition of the gut microbial community. **a**, **b** Alpha diversity metrics of the gut microbiota across experimental groups. **c** PCA of the gut microbiota in each group. Each dot represents an individual sample; color-coded dots indicate samples from different groups. The *x*-axis displays the first principal component, and the percentage represents its contribution to sample differentiation. The *y*-axis shows the second principal component, and the percentage represents its contribution to sample differentiation. **d** ANOSIM assessing the gut microbiota composition across groups. “Between” refers to the beta-diversity distance calculated across all groups. An *R* value close to 1 indicates that inter-group differences are greater than intra-group variations. A *P* < 0.05 indicates statistically significant differences among groups. **e**, **f** Bar chart illustrating the taxonomic composition at both the phylum and family levels. Each species is represented by a distinct color, with the length of the corresponding color block indicating its relative abundance. **g** Heatmap depicting the relative abundance of 44 gut bacterial taxa enriched in group B. Red indicates upregulation of the associated pathway, while blue reflects downregulation

The non-redundant gene sequences were compared using National Center for Biotechnology Information (NCBI)-BLAST, revealing that the cat gut microbiota comprised five kingdoms, 64 phyla, 89 classes, 314 families, 1045 genera, and 4454 species. The alpha diversity of gut microbiota was significantly reduced in both group A and group B compared with group C, and group A exhibited a more pronounced reduction (Fig. 5a, b). Furthermore, group A demonstrated a significant alteration in the beta diversity of gut microbiota (Fig. 5c, d).

The dominant species of the gut microbiota were found to be *Bacteroidetes*, *Firmicutes*, *Proteobacteria*, and *Actinobacteria*. However, significant variations in their relative abundances were observed across the three groups (Fig. 5e). The composition of dominant bacteria in group A and group B showed significant differences at the family level compared with that of group C (Fig. 5f). *Prevotellaceae*, *Lactobacillaceae*, *Bifidobacteriaceae*, and *Succinivibrionaceae* were the dominant bacteria in group A, whereas *Bacteroidaceae*, *Lachnospiraceae*, *Selenomonadaceae*, and *Lactobacillaceae* were prevalent in group B (Fig. 5f).

#### Species-level changes in bacterial composition

The relative abundance of 289 species exhibited significant alterations in group A and group B compared with group C. Among the top 100 differential bacterial species, 44 exhibited increased abundance in group B, whereas their abundance was reduced in group A. These bacteria consisted of 16 members from *Bacteroidetes*, 10 members from *Firmicutes*, 5 members each from *Proteobacteria* and *Actinobacteria*, and 4 members from *Fusobacteria* (Fig. 5g). This finding suggested that *Bacteroidetes* and *Firmicutes* played crucial roles in oocyst development and excretion.

### Distinctive gut bacteria at the oocyst excretion stage

**Fig. 6:**
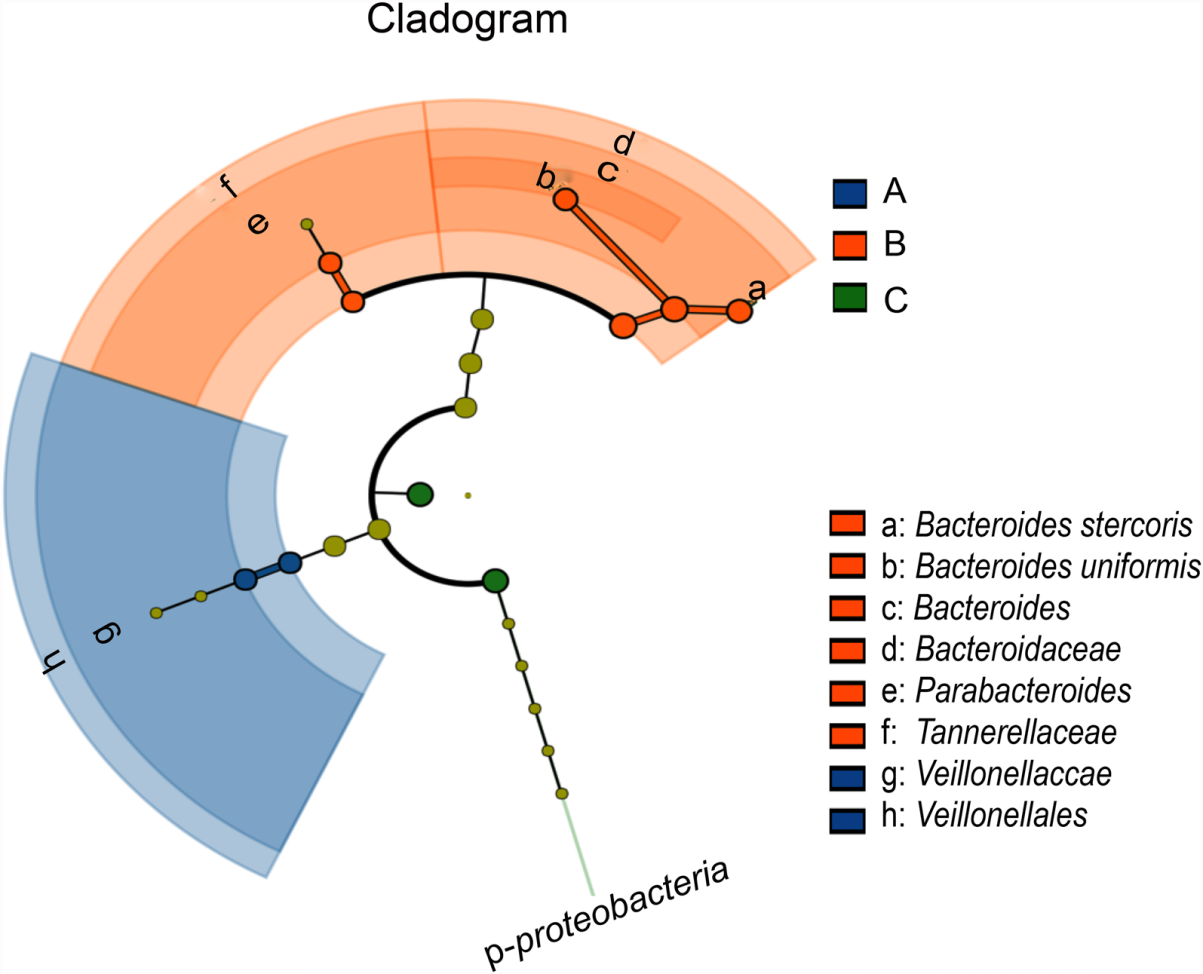
The LEfSe analysis of the gut bacterial communities among the three groups of cats. The circles radiating from the inside outward represent taxonomic levels ranging from phylum to species. At each classification level, each small circle represents a distinct category, with its size proportional to the relative abundance. Different colors correspond to different taxonomic groups

LEfSe was used to analyze bacteria that exhibited significant differences in group A and group B compared with the control group (group C). The results showed that *Veillonellaceae* from *Firmicutes* exhibited significant variation in group A, while *Bacteroides* within *Bacteroidaceae* likely played a crucial role in group B. *Bacteroides stercoris* displayed the most pronounced changes in group B, highlighting its importance during the *Toxoplasma* oocyst excretion stage and potentially serving as key bacteria during this stage (Fig. 6).

### Potential functional analysis of *Bacteroides stercoris* during the oocyst excretion stage

During the oocyst excretion stage (in group B)*, Bacteroides stercoris* demonstrated significant enrichment in seven KEGG pathways and 14 CAZymes compared with group C and group A. The Spearman correlation test was calculated on the basis of the differences in abundance, with selection criteria set at *|r|*> 0.7 and *P* < 0.05. The findings revealed significant associations both in KEGG pathways and CAZymes (Table 2). Notably, CBM6 was significantly associated with all five signaling pathways; meanwhile, CBM4, GH11, GH51, GH97, and GH115 exhibited significant associations with four of those pathways. Consequently, *Bacteroides stercoris* may play key roles in the *Toxoplasma* oocyst excretion by influencing the expression of CBM6, CBM4, GH11, GH51, GH97, and GH115 to regulate critical related pathways, including polyketide sugar unit biosynthesis, riboflavin metabolism, and lysine degradation. These findings may provide promising targets for developing strategies to block oocyst excretion, although further studies are needed to clarify the regulatory mechanisms.

**Table 2:** Correlation analysis between KEGG pathways and CAZymes in *Bacteroides stercoris*

KEGG pathways	Correlated CAZymes
Pantothenate and CoA biosynthesis	CBM6, CBM48, GH115
Polyketide sugar unit biosynthesis	CBM4, CBM6, GH26, GH43, GH51, GH97, GH115
Riboflavin metabolism	CBM4, CBM6, GH26, GH43, GH51, GH67, GH97, GH115
Lysine degradation	CBM4, CBM6, GH26, GH43, GH51, GH67, GH97, GH115
Phosphonate and phosphinate metabolism	CBM4, CBM6, GH19, GH51, GH97

## Discussion

*T. gondii* is recognized as the most widely distributed protozoan parasite globally. The oocysts of *T. gondii* play a crucial role in enhancing its transmissibility [30, 31]. Since the feline intestine is the sole site for the development and excretion of *Toxoplasma* oocysts, the research on functional and compositional changes in the feline gut microbiota during the sexual initiation and oocyst excretion stages of *T. gondii* has become urgent and relevant. The present study employed shotgun metagenomics to analyze the compositional and functional alterations in feline gut microbiota during the development and excretion stages of *T. gondii* oocyst, identifying a key bacterial species, *Bacteroides stercoris*, and relative functional factors. These findings provide mechanistic insights into the tripartite interaction among *T. gondii*, gut microbiome, and definitive host and may lay a foundation for future research into mechanisms and potential interventions.

Our results showed that *T. gondii* sexual initiation caused a significant downregulation of a large number of CAZymes genes spanning GHs, CBMs, and PLs—key mediators of mucosal barrier maintenance and immune evasion [32–35]—suggesting that the sexual initiation of tachyzoites may make the intestinal mucosal barrier more vulnerable and help parasites quickly invade intestinal epithelial cells. Intriguingly, the oocyst excretion phase exhibited a counteractive microbial adaptation, marked by the upregulation of approximately 40 CAZymes members predominantly involved in mucin glycan processing. Meantime, we also discovered that many VFs were significantly enriched at this stage. Particularly, among them, *iucC* and *shuU* are related to iron metabolism. Iron metabolism in the gut reduced host damage caused by redox reactions through bacterial siderophore-mediated iron sequestration and promoted probiotic bacteria abundance [36–38]. These variations suggest that the altered functional factors, such as CAZymes and VFs, may contribute to restoring the intestinal mucosal barrier and mitigating the gut damage induced by *T. gondii.*. In addition, the enrichment of antibiotic resistance genes (ARGs) against lincomycin and carbapenem at this stage can inhibit intestinal anaerobic bacteria [39, 40] and thereby potentially promote the excretion of oocysts. Similarly, this phenomenon has also been observed in mice infected with *Cryptosporidium*. After treating the infected mice with penicillin, the number of anaerobic bacteria in their intestines decreased. At the same time, the excretion of *Cryptosporidium* oocysts increased significantly [41].

Notably, the analysis of stage-specific key bacterial taxa revealed divergent microbial characteristics between the definitive and intermediate hosts. During the sexual initiation stage and oocyst excretion stage, cats exhibited significant increases in the relative abundance of *Firmicutes* and probiotic taxa, accompanied by a marked reduction in *Proteobacteria* abundance. This contrasts sharply with observations in *T. gondii*-infected mouse models (intermediate hosts) [42, 43], where these bacterial groups displayed opposite abundance patterns. Furthermore, when mice were infected with non-*Toxoplasma* parasites such as *Giardia* and *Cryptosporidium* [44, 45], the abundance variations of *Firmicutes* and *Proteobacteria* in their gut microbiota converged with those observed in *T. gondii*-infected mice. Collectively, these findings suggest that *T. gondii* exerts a unique stage-specific regulatory effect on the gut microbiota of the definitive host.

We further observed that the gut microbiota composition exhibited significant dynamic shifts across different developmental stages of *T. gondii*. During the sexual initiation stage, dominant bacterial families, including *Prevotellaceae*, *Lactobacillaceae*, *Bifidobacteraceae*, and *Succinivibrionaceae* may establish an immune-tolerant microenvironment for parasite sexual development via their anti-inflammatory properties and maintenance of intestinal mucosal barrier integrity [46–48]. When transitioning to the oocyst excretion stage, the microbiome underwent substantial structural remodeling, with the *Bacteroidaceae*, *Lachnospiraceae*, and *Selenomonadaceae* emerging as the new dominant taxa. Published studies indicate that these taxa possess multifaceted metabolic regulatory capacities such as (i) enhancing intestinal immune responses by promoting the biosynthesis of short-chain fatty acids (SCFAs), such as butyrate [50], and (ii) facilitating intestinal epithelial absorption and metabolism of linoleic acid (LA) [51]. Given that LA has been identified as a critical inducer of *T. gondii* sexual initiation [52], our findings collectively suggest that these dominant bacterial taxa may regulate oocyst excretion by modulating gut metabolites and reducing intestinal system damage. However, further metabolomic investigations are required to validate these mechanistic hypotheses.

Our results showed that *Bacteroides stercoris* may serve as a key mediator of *T. gondii* oocyst excretion, with its regulatory role potentially linked to the functional upregulation of CBMs and GHs. As a probiotic, *Bacteroides stercoris* exhibits multiple metabolic activities. It can not only promote the biosynthesis of SCFA [53] but also secrete heparinase and chondroitinase, which can degrade host-derived glycosaminoglycans [54, 55]. These observations suggest that *Bacteroides stercoris* may modulate oocyst excretion dynamics through dual mechanisms: (i) remodeling the intestinal metabolic milieu via SCFA-driven immunometabolic reprogramming and (ii) disrupting parasite–host interfacial structures through enzymatic degradation of critical adhesion molecules. However, its precise function requires further confirmation through in vitro co-culture studies, metabolite detection, and enzyme activity analysis. Only after these confirmations can we scientifically assess its potential as an additive in cat food to prevent toxoplasmosis in both pet and stray cats.

Although the present study has achieved enlightening results, with the use of shotgun metagenomics, coupled with the functional annotations (KEGG, CAZy, CARD, and VFDB), ensuring a comprehensive analysis of microbial functional and compositional changes, there are still inevitable limitations. First, the restricted sample size (*n* = 3 per group) may limit the statistical power and generalizability. Given this, we have established strict environmental controls, including standardized diets and SPF housing, to minimize confounding variables. In the following study, we will enlarge the sample size to enhance the statistical power. The integrated multi-omics strategy combining transcriptomics and targeted metabolomics simultaneously links gene function annotation with metabolic output, further exploring the dynamic interactions between the definitive host, the gut microbiota, and *T. gondii.*

## Conclusions

Overall, our results indicate that *T. gondii* manipulates the cat gut microbiota through a unique mechanism to regulate oocyst development. It may manipulate the cat gut microbiota to repair the intestinal mucosa barrier and reduce damage to the intestinal system, thereby protecting intestinal epithelial cells to promote oocyst excretion. *Bacteroides stercoris* may serve as a principal bacterium and play a crucial role in oocyst excretion by regulating key candidates of CBMs and GHs, making it a promising candidate for investigating a regulatory strategy for controlling oocyst excretion; however, the mechanisms still need further verification in larger sample sizes.

## Data Availability

The raw metagenomic data on cat gut microbiota of this article can be found online at: https://www.ncbi.nlm.nih.gov/sra/PRJNA1206810.

## Abbreviations

KEGG: Kyoto Encyclopedia of Genes and Genomes
CAZymes: Carbohydrate-active enzymes
VFs: Virulence factors
LA: Linoleic acid
*T.** gondii*: *Toxoplasma gondii*
*TgCtwh6*: Wh6, a Chinese I × II hybrid genotype strain of *T. gondii*, isolated from a stray cat in Wuhan, China
SPF: Specific pathogen-free
KM: Kunming
IECs: Intestinal epithelial cells
GO: Gene ontology
CAZy database: Carbohydrate-Active Enzymes database
CARD Database: Comprehensive antibiotic database
VFDB: Virulence factors of pathogenic bacteria
AROs: Antibiotic resistance ontologies
Nr: Non-redundant protein database
PCA: Principal coordinates analysis
ANOSIM: Analysis of similarities
LEfSe: Linear discriminant analysis Effect Size
GHs: Glycoside hydrolases
CBMs: Carbohydrate-binding modules
PLs: Polysaccharide lyases
GTs: Glycosyltransferases
SCFA: Short-chain fatty acids
